# An organic array of quantum corrals modulated by the gold herringbone electronic superlattice†

**DOI:** 10.1039/d5nr00148j

**Published:** 2025-03-18

**Authors:** Jun Li, Ignacio Piquero-Zulaica, Stefano Gottardi, Mustafa A. Ashoush, Zakaria M. Abd El-Fattah, Leonid Solianyk, Jose Enrique Ortega, Johannes V. Barth, Juan Carlos Moreno-Lopez, Jorge Lobo-Checa, Meike Stöhr

**Affiliations:** a Zernike Institute for Advanced Materials, University of Groningen, 9747 AG Groningen Nijenborgh 3 Netherlands m.a.stohr@rug.nl; b Centro de Física de Materiales CSIC/UPV-EHU Manuel Lardizabal 5 20018 San Sebastian Spain; c Physics Department E20, TUM School of Natural Sciences, Technical University of Munich James-Franck-Straße 1 D-85748 Garching Germany; d IKERBASQUE, Basque Foundation for Science Plaza Euskadi 5 48009 Bilbao Spain; e Physics Department, Faculty of Science, Al-Azhar University Nasr City E-11884 Cairo Egypt z.m.abdelfattah@azhar.edu.eg; f Physics Department, Faculty of Science, Galala University New Galala City Suez 43511 Egypt; g Departamento de Física Aplicada I, Universidad del País Vasco 20018 San Sebastian Spain; h Donostia International Physics Center (DIPC) Paseo Manuel de Lardizabal 4 E-20018 Donostia-San Sebastian Spain; i Institute of Solid State Physics, Vienna University of Technology 1040 Vienna Austria; j Instituto de Nanociencia y Materiales de Aragón, CSIC-Universidad de Zaragoza E-50009 Zaragoza Spain jorge.lobo@csic.es; k Departamento Física de la Materia Condensada, Universidad de Zaragoza E-50009 Zaragoza Spain; l University of Applied Sciences of the Grisons Pulvermühlestrasse 57 7000 Chur Switzerland

## Abstract

The periodic herringbone reconstruction on the surface of Au(111) consists of alternating face-centered-cubic (fcc) and hexagonal-closed-packed (hcp) sites separated by dislocation lines and elbows. This well-known arrangement acts as an electronic superlattice for surface-state electrons, creating a mini-gapped band structure with a modulated electronic density. This rich and fascinating geometrical and electronic landscape has countless times served as a platform for molecular self-assembly and on-surface synthesis of carbon-based nanoarchitectures as well as a template for 2D material growth. In this work, we fabricated a long-range ordered organic quantum corral (QC) array *via* the self-assembly of 1,3,5-benzenetribenzoic acid molecules onto the herringbone reconstructed Au(111) surface. The periodicity of this QC array is nearly half the one of the underlying Au herringbone reconstruction, enabling us to study the delicate interplay between the two potential landscapes by allowing the selective formation and electronic modulation of QCs both on hcp and fcc sites. Scanning tunneling microscopy/spectroscopy (STM/STS) can probe such local differences in the first partially confined state and finds that not only the energy onset of the surface state electrons is influenced but also the modulation of the shallow herringbone potential contributes to the newly formed band structure. This is confirmed by angle-resolved photoemission spectroscopy (ARPES), where the interplay of the periodic potentials introduced by the organic QC array and herringbone reconstruction results in the formation of a distinct surface state band structure. These results are corroborated and intuitively understood with electron-plane-wave expansion (EPWE) simulations. Our work shows that combined molecular and non-organic patterning can serve as a promising tool to macroscopically tune the electronic properties of metal surfaces in a controllable manner.

## Abstract 2

We unveil the modulation of the electronic structure induced by the delicate interplay between the Au(111) herringbone potential and that of an organic array of quantum corrals.

## Graphical abstract

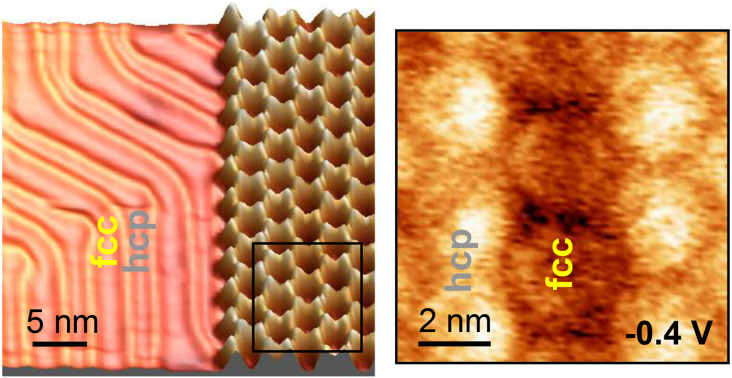

## Introduction

Surface reconstruction refers to the lateral and/or vertical rearrangement of surface atoms that minimizes the surface free energy. This leads to two-dimensional changes in the structure of the surface layer.^1^ In surface science, this is a commonly observed effect for transition metal and semiconductor materials, whereby the 7 × 7 reconstruction of Si(111)^2^ and the 22 × √3 herringbone reconstruction on Au(111)^3^ are seminal examples. The latter can be understood as a densification of the top layer every 22 lattice constants by inserting an extra Au atom leading to an additional [01̄0] atomic chain.^4–6^ This reconstructed gold surface is characterized by well-defined face-centered-cubic (fcc) and hexagonal-closed-packed (hcp) sites separated by dislocation lines (also interpreted as solitons) and elbow dislocations. The Au herringbone reconstruction influences many applications of Au(111), for example by templating molecular self-assemblies,^7,8^ enabling on-surface synthesis of carbon-based nanostructures such as graphene nanoribbons,^9,10^ nanographenes^11^ and nanoporous graphene structures,^12,13^ or participating in the growth of low-dimensional materials *e.g.*, transition-metal dichalcogenides^14^ (TMDs), transition-metal dihalides^15^ (TMHs), carbon nitride,^16^ 2D metal–organic networks,^17,18^ or simply by allowing to explore the physical or chemical properties of various molecular or atomic absorbates.^19^ Notably, the long-range herringbone reconstruction acts as an electronic superlattice for surface-state electrons, creating a new featured band structure with a modulated electron density. Using an extended Kronig–Penney model, it was estimated that the electronic potential energy difference between the fcc and hcp regions of the reconstruction, responsible for the superlattice band structure, is 25 ± 5 meV.^20^ The herringbone reconstruction can be altered (even quenched) by tuning its chemical interaction with single atoms or molecular absorbates,^21,22^ as well as by introducing periodically spaced steps on vicinal surfaces.^23^

Another way of interacting with the existing surface potential consists of fabricating potential cages, also known as quantum corrals (QCs). Initially, QCs^24,25^ were fabricated *via* scanning tunneling microscopy (STM) through the tedious approach of atom manipulation. The surface state electrons were confined within these closed structures, which were conceptually conceived as artificial quantum dots or atoms.^26^ The periodic repetition of these QCs into an ordered array on top of the herringbone reconstruction would then allow the study of the modulation of the QCs's electronic properties by the underlying gold electronic superlattice at both the local and mesoscopic scales. Although the STM manipulation method offers ultimate precision in building such artificial nanostructures at the local scale,^27–32^ it is impractical when constructing significantly large arrays on the surface. A less demanding approach exists that takes advantage of the molecular and metal–organic self-assembly processes and that offers the possibility to fabricate extensive, well-ordered molecular nanostructures on surfaces with ease.^33–35^ In this way, periodic scattering potential landscapes based on organic 2D nanoporous networks have been successfully bestowed to different metal surfaces with the ability to confine the surface electrons, ultimately modifying their band structure.^36–38^ Similar to QCs, each nanopore acts as an artificial atom or quantum dot, which no longer behaves independently from its adjacent neighbours.^38^ By using molecular building blocks with different sizes and geometries, as well as directional metal–organic bonds, the pore size and the symmetry of the porous networks can be adjusted, which in turn tunes the electronic structure of the entire surface in a controllable way.^17,36,38,39^

In this study, we used the molecule 1,3,5-benzenetribenzoic acid (BTB) to synthesize an atom-thick organic nanoporous network by direct deposition on Au(111). We chose this molecule since it is known to form well-ordered open honeycomb network structures on Ag(111), graphite and graphene, with a periodicity of nearly half the size of the herringbone lattice.^40–42^ Such periodicities are key to studying the interplay between the inherent weak surface potential associated with the reconstructed surface of Au(111) and the periodic, presumably stronger, potential induced by the molecular network. As shown by STM, BTB molecules are arranged in a hydrogen-bonded honeycomb lattice on Au(111). This long-range, well-ordered nanoporous array (or array of coupled QCs) exhibits a clear diffraction pattern in low-energy electron diffraction (LEED) measurements and does not alter the herringbone reconstruction, implying a rather weak interaction between the molecules and the Au surface. Scanning tunneling spectroscopy (STS) performed at 4.5 K showed that the surface state electrons of Au(111) are restricted to discrete energy levels due to quantum confinement in each nanopore of the network, the first of which is selectively modulated by the herringbone electronic superlattice hosting fcc and hcp sites. We found that the finite scattering barriers exerted by the molecules promoted the coupling of QCs and induced the formation of a distinct band structure with zone boundary gap openings, as observed with ARPES. These results are supported and intuitively understood by semiempirical electron plane-wave expansion (EPWE) simulations.

## Results

**Fig. 1:**
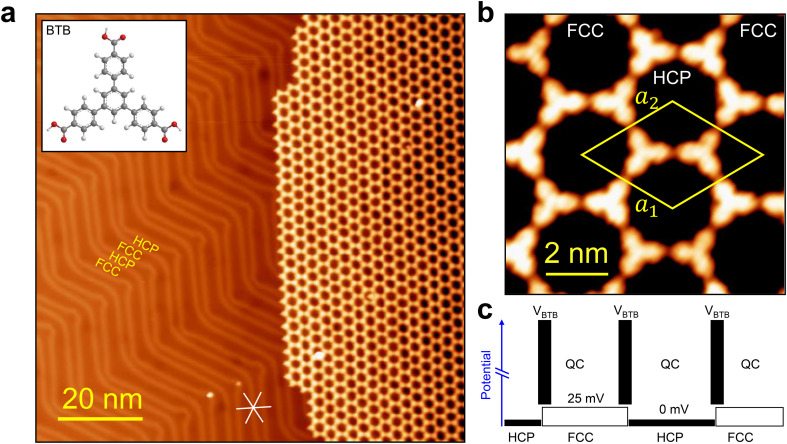
BTB organic quantum corral array formation on Au(111). (a) STM image corresponding to sub-monolayer coverage of BTB after post-deposition annealing at 400 K. The BTB molecules exclusively arranged in a honeycomb lattice over the herringbone reconstruction of the substrate (*V* = −1 V; *I* = 20 pA). Inset: structure of 1,3,5-benzenetribenzoic acid (BTB) composed of carbon (gray), oxygen (red) and hydrogen (white) atoms. (b) Detailed STM image of the honeycomb network, where each molecule is visualized as a three-fold clover and the unit cell is marked in yellow (*V* = −700 mV; *I* = 1.2 nA). (c) Schematic surface potential landscape of the organic quantum corral array and the herringbone heterostructure. The shallow electronic potential of the herringbone reconstruction modulates the electronic properties of the overlying organic quantum corral array (*V*_BTB_).

The employed BTB molecule (Fig. 1a, inset) has a symmetric three-fold shape and three terminal carboxylic acid groups. Upon deposition of BTB on Au(111) held at room temperature and subsequent post-deposition annealing at 400 K for 30 minutes, a well-ordered hexagonal porous network is formed stabilized *via* intermolecular hydrogen bonding between adjacent carboxylic acid groups (Fig. 1a). Remarkably, we found the domain sizes to exceed 100 nm, being only restricted by the Au terrace size they grew on (see Fig. S1 in the ESI†). As intended, the presence of the nanoporous network does not disturb the underlying herringbone reconstruction, forming an atomically well-defined heterostructure. Indeed, the features of the herringbone reconstruction were distinguishable through the nanoporous network in many STM images (see Fig. S1 in the ESI†).

The long-range order of the nanoporous network was confirmed by LEED measurements (Fig. S2 in the ESI†). From the diffraction pattern, the network is found to exhibit a relative rotation of 30° ± 3.5° with respect to the principal directions of the Au(111) substrate. From the detailed STM image with intramolecular resolution (Fig. 1b), we infer that the molecules are arranged in a honeycomb lattice with each hexagonal pore defined by six molecules. The rhombic unit cell (indicated in yellow) has an average size of *a*_1_ = *a*_2_ = 3.28 ± 0.14 nm and an opening angle of *θ* = 60° ± 0.5°. The molecules lie flat on the Au(111) surface (disregarding a slight possible rotation of the phenyl rings) and each molecule undergoes double H-bonding with three neighboring molecules *via* the carboxylic endgroups. The combination of intermolecular double H-bonds between adjacent molecules and molecule–substrate interactions is responsible for stabilizing the long-range ordered network, similar to previous reports on Ag(111)^40^ and graphene/Cu(111).^42^ Interestingly, the BTB nanoporous network exhibits a periodicity of nearly half the one of the herringbone reconstruction (3.28 nm *vs.* 6.34 nm). This is fundamental to this work as certain QCs can be found strictly in registry with either fcc or hcp stacking areas (Fig. 1b). In consequence, the electronic superlattice defined by the herringbone reconstruction should both locally and macroscopically modulate the electronic properties of the organic QC array effectively creating a heterostructure potential landscape (Fig. 1c). Note that due to the non-perfect commensurability of the BTB QC array, some nanopores are inevitably positioned in between fcc and hcp regions.

**Fig. 2:**
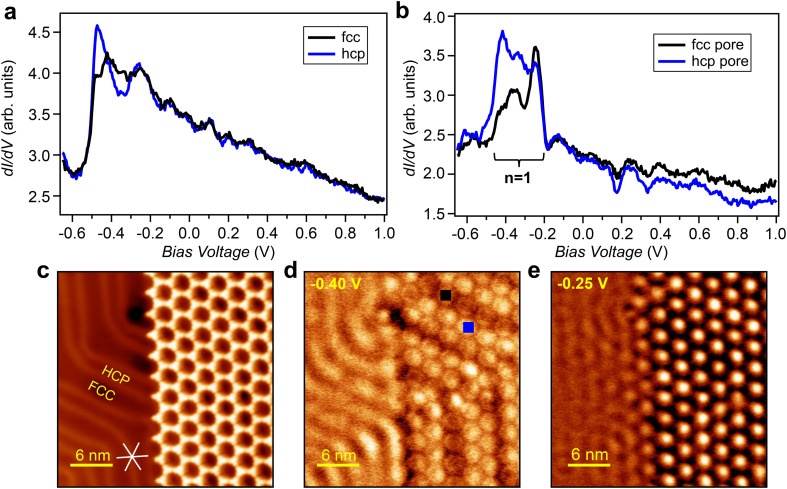
Scanning tunneling spectroscopy on the pristine herringbone reconstructed Au surface and on the QC array/herringbone heterostructure. (a) Tunneling spectra obtained on the hcp (blue) and fcc (black) sites of the bare Au(111) substrate (*V*_set_ = −700 mV; *I*_set_ = 90 pA). (b) STS taken in the center of a bright pore located on an hcp area (blue spectrum, position marked in (d) with a blue square) and a dark pore located on an fcc area (black spectrum, position marked in (d) with a black square) (*V*_set_ = −700 mV; *I*_set_ = 100 pA). The *n* = 1 partially localized state is indicated with a bracket. (c) STM image for sub-monolayer coverage of BTB on Au(111) after annealing at 400 K (*V* = −250 mV; *I* = 90 pA). (d and e) Constant current d*I*/d*V* maps taken at the same position as (c) and acquired at −0.40 V (*I* = 90 pA) and −0.25 V (*I* = 90 pA), respectively.

To investigate the effect of the modulation of the herringbone electronic superlattice on the BTB QC array, STS measurements were performed (at 4.5 K) in a region where both the pristine Au(111) surface and the porous network coexisted (Fig. 2c). The tunneling spectra acquired at the fcc and hcp sites of the bare Au(111) surface are displayed in Fig. 2a and serve as a reference for the measurements performed in the areas covered by the BTB network. Electronic differences are visible between the two regions of the pristine substrate: the spectrum at the hcp zone (in blue) exhibits a sharp vertical increase at the surface state onset (at ≈−0.48 V), whereas the spectrum taken at the fcc zone (in black) shows comparably weaker amplitude at the onset. Additional intensity modulations occur between −0.45 V and −0.2 V, while for higher energies, both signatures become practically identical. These local density of states (LDOS) differences are known to be caused by the periodically varying surface potential of the Au(111) herringbone reconstruction.^20^ Notably, such line shape variations are perfectly captured in our EPWE LDOS simulations of the pristine Au(111) surface (see Fig. S3 in the ESI†).

Turning to the network region, the potential induced by the molecules is now expected to confine the Au surface state electrons inside the nanopores.^36^ Following the pristine case, we distinguish the two local sites (fcc and hcp pores) when performing STS measurements at the nanopore centers (see Fig. 2b). While for the STS spectra obtained at the hcp pore center (in blue), the entire region between −0.5 V and −0.25 V showed (almost plateau-like) high intensity, the LDOS at the fcc pore center (in black) exhibited two peaks located around −0.4 V and −0.25 V. The distinct features observed inside each pore below −0.25 eV reflect a noticeable interplay with the underlying herringbone reconstruction, which despite its weak surface potential considerably influences the electronic features inside the nanopores. Note that such local differences are generally absent for other explored organic QC arrays grown commensurably on unreconstructed surfaces such as Ag(111) and Cu(111) when the pores are identical and do not present defects.^36^

To examine the spatial distribution of the LDOS, constant current d*I*/d*V* maps were acquired at −0.4 V (Fig. 2d) and −0.25 V (Fig. 2e). The map at −0.25 V exhibits single dome-like features at the center of each nanopore, which is a typical signature for a first resonance (*n* = 1) in surface state quantum confinement within coupled QCs.^17,37,38,43^ In contrast, for −0.4 V, only a striped pattern both on the bare Au surface and on the molecular network covered areas was observed. Notably, this state seems to be rather insensitive to the supramolecular overlayer periodicity. By comparing Fig. 2c and d, it becomes evident that the dark stripes observed in the d*I*/d*V* map are located on fcc zones while the bright ones are located on the hcp zones and spread slightly over the dislocation lines. Thus, the striped pattern has a close relationship with the herringbone reconstruction of Au(111) and is unrelated to the QC array potential landscape. It has been reported that d*I*/d*V* maps acquired on bare Au(111) at −0.48 eV also exhibited a similar striped pattern due to the difference in intensities of the LDOS in the fcc and hcp zones of the reconstructed surface.^20^ In response to this potential superlattice, the low-energy surface state electrons tend to be localized in the hcp regions of the reconstructed Au(111) surface, while the trend is reversed for the electrons with slightly higher energy (*i.e.*, above the herringbone gap at the zone boundary), shifting the LDOS maxima to the fcc regions of the reconstructed surface. Indeed, this intensity inversion matches our STS point spectra shown in Fig. 2a as well as the EPWE LDOS simulations (Fig. S3 in the ESI†). We note that extra intensity modulations are visible at the elbows of the herringbone reconstruction. However, they are extremely site dependent and inhomogeneous within a single pore of the organic network. Such a complex potential reconfiguration of the substrate prevents it from being periodic with respect to the BTB network. Thus, pores at the elbow positions are intentionally discarded in this work.

**Fig. 3:**
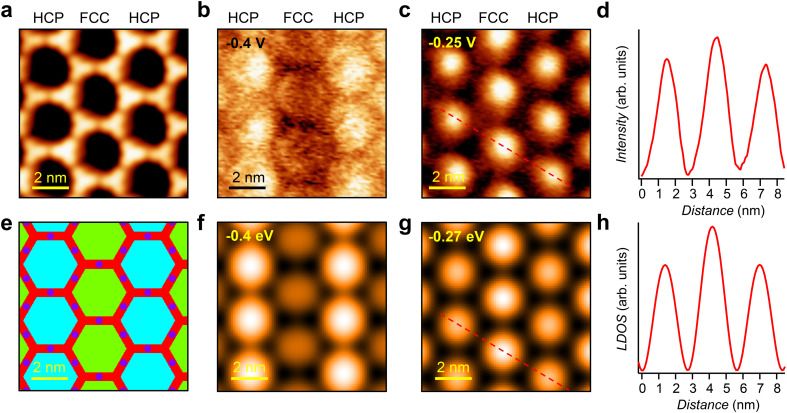
d*I*/d*V* and LDOS maps for the *n* = 1 state of the hcp and fcc quantum corrals. (a) STM image of the porous network (*V* = −250 mV; *I* = 90 pA). Constant-current d*I*/d*V* maps taken at the same position as (a) at (b) −0.4 V (*I* = 90 pA) and (c) −0.25 V (*I* = 90 pA). (d) The conductance intensity line profile traversing the hcp-fcc-hcp pores taken along the dashed line in (c). The conductance dominates at the fcc site. The line profile has been averaged over 10 hcp-fcc-hcp pore segments (see Fig. S4†). (e) Constructed surface potential landscape (*V* = 0 V turquoise, *V* = 0.025 V green, *V* = 0.275 V red, and *V* = 0 V dark blue) of the BTB network/herringbone heterostructure used for the EPWE calculations. From this landscape, the EPWE simulated spatial distribution of the LDOS was done at (f) −0.4 eV and (g) −0.27 eV. (h) The LDOS intensity line profile traversing the hcp-fcc-hcp pores taken along the dashed line in (g). All simulated data are in excellent agreement with the experimental ones.

To gain insight into the underlying mechanism of such striped patterns, we calculated the LDOS at the herringbone/QC array interface with EPWE (see the Methods section). The scattering potential landscape for these calculations is shown in Fig. 3e and is based on the STM data (see Fig. 3a). The potential difference between the fcc and hcp regions is estimated to be 25 mV, following previous studies.^20^ Therefore, we assign a potential of 25 mV to the fcc zones and a potential of 0 mV to the hcp ones. For the BTB porous network, we assign a molecular scattering potential of 275 mV and 0 mV to the intermolecular double hydrogen-bonding sites, based on similar systems showing electron transmission channels.^39,44^ The onset of the Au Shockley surface state is set to −0.5 eV and the effective mass to 0.26 m_e_.^45^Fig. 3f shows the calculated LDOS at −0.4 eV, whereby the fcc zone appears darker (lower LDOS) than the hcp one (higher LDOS). Notably, these calculations nicely reproduce the striped pattern with alternating brightness obtained in the d*I*/d*V* map at −0.40 V (Fig. 3b), providing confidence in our theoretical approach.

To understand the spatial distribution of the surface state electrons at higher energy (see the d*I*/d*V* map taken at −0.25 V in Fig. 3c), the LDOS is also calculated at −0.27 eV (see Fig. 3g). In this case, not only the confinement of the surface state into each QC (dome shape in the pores) is nicely captured, but also the fcc zone exhibits a slightly higher LDOS intensity than the hcp one (*i.e.*, an inverted brightness compared to the LDOS at −0.4 V). This is clearly visualized in the line profiles shown in Fig. 3d and h (extracted from Fig. 3c and g, respectively) which indicates that the LDOS intensity is higher at the fcc QCs than at the hcp QCs (see Fig. S4†). As will become evident later, this intensity inversion at −0.25 V (top of the *n* = 1 partially localized state) compared to the −0.4 V (bottom of the *n* = 1 partially localized state) d*I*/d*V* map is related to the fact that at −0.4 V/−0.25 V, one is energetically below/above the herringbone induced band gap of the surface state band structure. Overall, it can be concluded that the surface state electrons are influenced by a combination of scattering potentials of the molecular network and the herringbone reconstruction (Fig. S3 in the ESI†). Thus, unlike coupled QC arrays grown on homogeneous surfaces [e.g., Ag(111)^38^ or Cu(111)^37^] which exhibit a single dome-like *n* = 1 state, here, this state is split in energy due to the herringbone potential leading to local intensity variations.

**Fig. 4:**
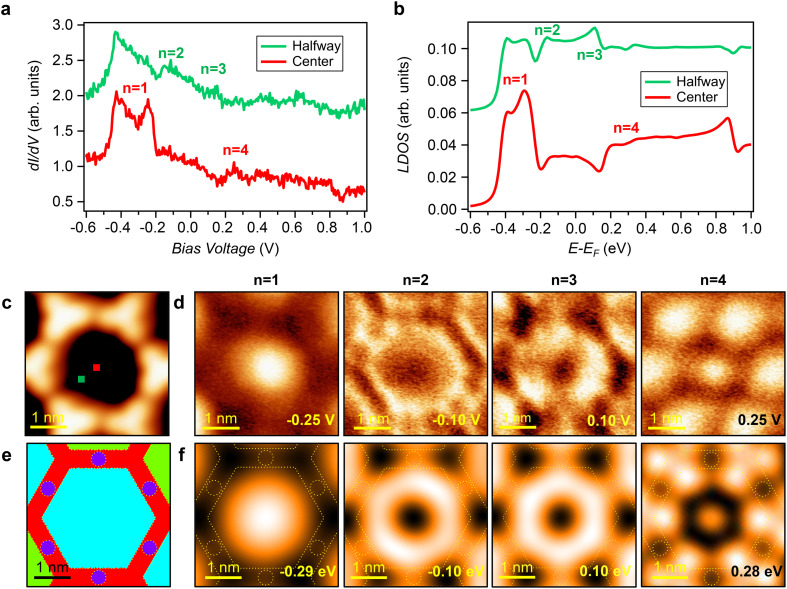
Surface state confinement in an hcp QC. (a) STS point spectra obtained at the center and halfway positions of the hcp QC (*V*_set_ = −700 mV; *I*_set_ = 90 pA). The positions at which the STS spectra were acquired are indicated with a red and green square in (c). (b) EPWE simulated LDOS spectra at the center and halfway positions. (c) STM image of a single hcp QC (*V* = −250 mV; *I* = 90 pA). (d) Constant-current d*I*/d*V* maps taken at −0.25 V (*I* = 90 pA) (left), −0.10 V (*I* = 90 pA) (middle-left), 0.1 V (*I* = 90 pA) (middle-right) and 0.25 V (*I* = 90 pA) (right), corresponding to the first, second, third and fourth confined states. (e) Surface potential landscape used in EPWE, considering a molecular scattering potential of 275 mV for the red regions, 0 mV for the dark blue and for the turquoise hcp potential. (f) EPWE simulated spatial distributions of the LDOS at −0.29 eV (left), −0.1 eV (middle-left), 0.1 eV (middle-right) and 0.28 eV (right) matching the corresponding experimental cases.

To further examine the confinement of the Au surface state in the nanopores of the BTB network, we focused on a QC located at an hcp zone (Fig. 4). STS measurements taken at the center and halfway between the center and the nanopore edge (Fig. 4a) showed local maxima (besides the one at −0.4 eV) at energies of −0.25 V (center position), −0.1 V (halfway position), around 0.1 V (halfway position) and +0.25 V (center position). We acquired d*I*/d*V* maps at these four energies to determine the spatial distribution of these states. At −0.25 V (Fig. 4d, left), we identified a dome-like shaped protrusion with its maximum in the center of the pore, which corresponds to the ground state energy of the first confined state (*n* = 1). By increasing the energy to −0.1 V (Fig. 4d, middle-left), the LDOS exhibited a donut shape with the highest intensity located between the pore center and its rim, which agrees with the theoretical spatial distribution of the second confined state (*n* = 2). The *n* = 3 confined state is similar to *n* = 2 and was identified at 0.1 V (Fig. 4d, middle-right). Finally, the d*I*/d*V* map taken at 0.25 V (Fig. 4d, right) showed a more intricate structure than the previous three, where in addition to the central maximum, six intense features emerged at the positions of the BTB molecules. We simulated the experimental data by EPWE using the same scattering parameters as before (comparing Fig. 3e and 4e). Both the experimental STS point spectra (Fig. 4a) and LDOS features of the d*I*/d*V* maps (Fig. 4d) are nicely reproduced by our calculations (see Fig. 4b and f), confirming the top of the *n* = 1 state at −0.25 V, the *n* = 2 at −0.10 V, the *n* = 3 at 0.1 V and identifying the state at +0.25 V as the spatial distribution of the fourth confined state (*n* = 4). Moreover, experiments and simulations are in good agreement when comparing the line d*I*/d*V* spectra taken in two different directions through the BTB pore (either starting and finishing at the centers of the pore sides or at the corners), which further highlight the spatial distribution of the different confined states (Fig. S5 and S6 in the ESI†).

To understand how the herringbone reconstruction modulates the energetic position of the different confined states within the coupled QCs, we compare the simulated fcc QC sites with the ones corresponding to the hcp sites (Fig. S6 in the ESI†). Despite the weak herringbone potential (25 mV), we detected intensity modulations both at the bottom and at the top of the *n* = 1 state, as well as a tiny energy shift. This is caused by the presence of the herringbone minigap (15 mV) slightly above the bottom of the *n* = 1 state. Thus, the herringbone modulation affects the electronic structure only in the vicinity of this state, leaving the higher-in-energy confined states (*i.e.*, *n* = 2, 3 and 4) mostly unaffected by the underlying herringbone electronic superlattice (see Fig. S3 and S6 in the ESI†).

**Fig. 5:**
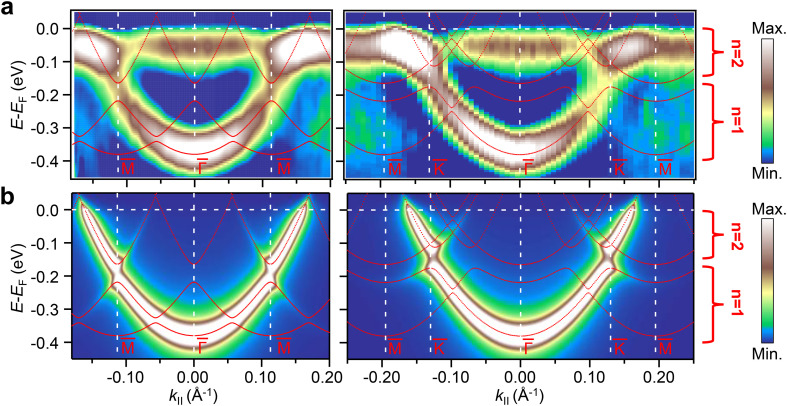
ARPES band structure in comparison with EPWE simulations of the Au surface fully covered with the BTB molecular network. (a) ARPES measurements taken along the 
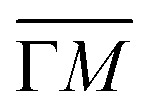
 (left) and 
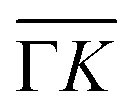
 (right) directions. The experimental plots present the second derivative of the energy dispersion curves to enhance the weak gap openings. (b) EPWE calculated band structure and photoemission intensities of the Shockley surface state taken along the 
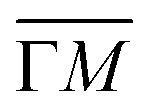
 (left) and 
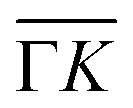
 (right) directions. All panels include superimposed in red the EPWE calculated band structure of the modified Shockley surface state, and vertical white lines indicating the symmetry points. The color scale and the energy range of the first two confined states are indicated on the right of the figure.

The question remaining is how this local influence on the QC array affects the band structure of the system (*i.e.*, at the macroscopic level). To this end, ARPES measurements (at 150 K) were performed on a sample with the organic network fully covering the surface. Fig. 5a shows the second derivative of the ARPES data plotted along the 
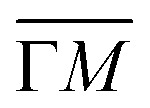
 (left) and 
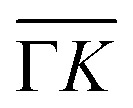
 (right) directions of this coupled QC array. The confinement of the pristine surface state gives rise to a new derived dispersive band related to the coupling of neighboring QCs (*i.e.*, induced by the leaky confinement through the molecular potential barriers^37^). The band bottom of the *n* = 1 partially localized state is detected around −0.4 eV, which is a ≈75 meV shift compared to the pristine Au(111) case (see Fig. S7 in the ESI†). This is in reasonable agreement with the EPWE simulations that show a shift of ≈70 meV. It is notable that the dispersion of the new band is relatively broad compared to the results reported in previous studies.^36^ However, this broadening in ARPES can be attributed to the combined contribution of the two mirror domains of the BTB network detected by LEED measurements (relative rotation of 30° ± 3.5° with respect to the principal Au(111) substrate directions, Fig. S2 in the ESI†). Note that with our limited instrumental resolution and presence of two domains, the spin–orbit splitting of the pristine surface state apparently vanishes (compare with Fig. S7 in the ESI†), but this does not imply its absence as it could be simply masked by the ARPES lineshape broadening.^17^ Despite this broadening, band gap openings are still discernible at ±0.11 Å^−1^ along the 
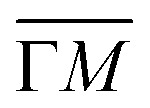
 direction and ±0.13 Å^−1^ along the 
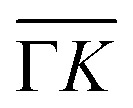
 direction (reduced intensity at the zone boundaries) and band replicas at higher momenta. These *k* points match the new periodicity introduced by the BTB network. Therefore, these band gap openings can be assigned to the periodic potential induced by the organic QC array.

The modified surface-state band structure by this QC array/herringbone heterostructure can also be simulated with EPWE (spin–orbit terms are not considered). As shown in Fig. 5b, the calculated band structure (red dotted lines, also superimposed in Fig. 5a) and photoemission intensities along the 
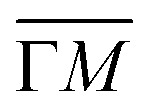
 (left) and 
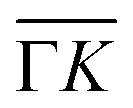
 (right) directions closely reproduce the experimental features of Fig. 5a. This comprises the band gap openings around the critical momentum and energy values, and the band replica at higher momenta. These zone boundary gaps allow us to identify the dispersive bands corresponding to the *n* = 1 and *n* = 2 partially localized states, energetically matching with the STS shown in Fig. 4. Furthermore, the calculated band structure along the 
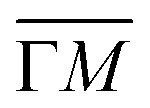
 direction also shows a small band gap opening around 0.05 Å^−1^ corresponding to the periodicity of the herringbone reconstruction of the Au(111) surface. Note that this gap is located within the *n* = 1 band. However, our limited experimental resolution hindered us from detecting such a tiny gap. We stress that this herringbone gap is not even observable in our pristine Au(111) surface state band measurements (Fig. S7 in the ESI†), in agreement with other reported ARPES measurements.^46–48^

## Conclusion

In summary, we fabricated with BTB molecules a long-range ordered organic QC array on Au(111) while preserving the herringbone reconstruction of the substrate. The nearly half-matching achieved between both lattices turns out to be key to study the delicate interplay between two potential landscapes of distinct nature and their impact on the electronic quantum confinement at the nanoscale. Indeed, the surface electronic structure is altered both due to the inherent surface potential associated with the reconstructed Au(111) surface and the additional periodic potential induced by the organic QC array. Notably, the QCs formed by the organic layer are modulated by the herringbone electronic superlattice and exhibit local differences depending on their fcc or hcp site location. Close to the energy minima (below the herringbone electronic gap), the low energy surface electrons are predominantly confined into the hcp stripes, while above this tiny gap this effect is reversed. The organic QCs show the expected discrete confined states, the first of which (*n* = 1) is modulated by the herringbone shallow potential.

These findings allow us to envision preferential adsorption of guest species (*e.g.*, additional atoms or molecules) depending on the underlying fcc or hcp sites that spatially define the QCs. Overall, our study shows that a combination of different patternings offers the possibility of not only macroscopically tuning the overall electronic surface properties, but also fine-tuning and varying the microscopic properties.

## Methods

### Electron plane wave expansion (EPWE)

The electron-plane-wave-expansion (EPWE) calculations were performed for the pre-defined potential landscapes shown in Fig. 3(e) corresponding to the BTB nanoporous network. The potential inside the hexagonal hcp (green) and fcc (turquoise) pores was set to 0 and 25 meV, respectively, while the potential at the honeycomb molecular backbone (red) was kept at 275 meV. The potential at the intermolecular double hydrogen bonds (dark blue) was set to zero following previous work on similar systems showing electron transmission channels.^39,44^ The reference energy was chosen at the Au(111) surface state onset, *i.e.*, −0.5 eV and −0.45 eV for STS and ARPES measurements, respectively, and the effective mass was kept at 0.26 m_e_. The Schrödinger equation was then solved for these potential landscapes.^49^ To achieve high convergence, the potential expansion was terminated at *g*_max_ = 7, where *g*_max_ refers to the window of the reciprocal lattice vectors sampled in the calculations.

### Experimental details

The STM measurements were performed in a two-chamber ultrahigh vacuum (UHV) system (base pressure ≈ 10^−11^ mbar) with a low temperature STM (Omicron Nanotechnology GmbH) operated at 4.5 K. The STS measurements were performed using a lock-in amplifier with a modulation amplitude of 10 mV (rms) and a frequency of 678 Hz. The STM images were analyzed with the WSxM software.^50^ The given bias voltages refer to a grounded sample.

The ARPES measurements were performed in a second UHV system (base pressure of 1 × 10^−10^ mbar) with a display-type hemispherical electron analyzer (SPECS Phoibos 150), an energy/angle resolution of 40 meV per 0.1° and a monochromatized Helium I (*hν* = 21.2 eV) source. The sample temperature during ARPES measurements was set to 150 K.

A clean and flat Au(111) substrate was prepared by repeated cycles of Ar^+^ sputtering and subsequent annealing at 770 K. BTB molecules were sublimated *in situ* from a Knudsen cell (at 540 K) onto the Au(111) substrate held at room temperature.

## Author contributions

J. L.-C. and M. S. conceived this project. J. L., S. G., L. S., and J. C. M.-L. conducted STM/STS measurements. I. P-Z. and J. L.-C. performed ARPES measurements and data analysis. J. E. O. provided resources and supervision. M. A. A. and Z. M. A. conducted EPWE simulations. J. L., I. P-Z., Z. M. A., J. L.-C. and M. S. contributed to writing the manuscript. All authors contributed to the revision and final discussion of the manuscript.

## Conflicts of interest

The authors declare no competing financial interests.

## Supplementary Material

NR-017-D5NR00148J-s001

## Data Availability

The data supporting this article have been included as part of the ESI.†

## References

[cit1] Duke C. B. (1996). Semiconductor Surface Reconstruction: The Structural Chemistry of Two-Dimensional Surface Compounds. Chem. Rev..

[cit2] Binnig G., Rohrer H., Gerber C., Weibel E. (1983). 7 × 7 Reconstruction on Si(111) Resolved in Real Space. Phys. Rev. Lett..

[cit3] Barth J. V., Brune H., Ertl G., Behm R. J. (1990). Scanning tunneling microscopy observations on the reconstructed Au(111) surface: Atomic structure, long-range superstructure, rotational domains, and surface defects. Phys. Rev. B: Condens. Matter Mater. Phys..

[cit4] Li P., Ding F. (2022). Origin of the herringbone reconstruction of Au(111) surface at the atomic scale. Sci. Adv..

[cit5] Hanke F., Björk J. (2013). Structure and local reactivity of the Au(111) surface reconstruction. Phys. Rev. B: Condens. Matter Mater. Phys..

[cit6] Seitsonen A. P. (2016). Electronic structure of reconstructed Au(111) studied with density functional theory. Surf. Sci..

[cit7] Barth J. V. (2007). Molecular Architectonic on Metal Surfaces. Annu. Rev. Phys. Chem..

[cit8] Liu H. (2023). *et al.*, Condensation and asymmetric amplification of chirality in achiral molecules adsorbed on an achiral surface. Nat. Commun..

[cit9] Cai J. (2010). *et al.*, Atomically precise bottom-up fabrication of graphene nanoribbons. Nature.

[cit10] Houtsma R. S. K., Enache M., Havenith R. W. A., Stöhr M. (2022). Length-dependent symmetry in narrow chevron-like graphene nanoribbons. Nanoscale Adv..

[cit11] Mishra S. (2021). *et al.*, Large magnetic exchange coupling in rhombus-shaped nanographenes with zigzag periphery. Nat. Chem..

[cit12] Moreno C. (2018). *et al.*, Bottom-up synthesis of multifunctional nanoporous graphene. Science.

[cit13] Piquero-Zulaica I. (2024). *et al.*, Deceptive orbital confinement at edges and pores of carbon-based 1D and 2D nanoarchitectures. Nat. Commun..

[cit14] Silva C. C. (2022). *et al.*, Spatial variation of geometry, binding, and electronic properties in the moiré superstructure of MoS_2_ on Au(111). 2D Mater..

[cit15] Aguirre A. (2024). *et al.*, Ferromagnetic Order in 2D Layers of Transition Metal Dichlorides. Adv. Mater..

[cit16] Gammelgaard J. J. (2023). *et al.*, A Monolayer Carbon Nitride on Au(111) with a High Density of Single Co Sites. ACS Nano.

[cit17] Piquero-Zulaica I. (2019). *et al.*, Surface state tunable energy and mass renormalization from homothetic quantum dot arrays. Nanoscale.

[cit18] Gottardi S. (2014). *et al.*, Cyano-Functionalized Triarylamines on Au(111): Competing Intermolecular versus Molecule/Substrate Interactions. Adv. Mater. Interfaces.

[cit19] Madhavan V., Chen W., Jamneala T., Crommie M. F., Wingreen N. S. (2001). Local spectroscopy of a Kondo impurity: Co on Au(111). Phys. Rev. B: Condens. Matter Mater. Phys..

[cit20] Chen W., Madhavan V., Jamneala T., Crommie M. F. (1998). Scanning Tunneling Microscopy Observation of an Electronic Superlattice at the Surface of Clean Gold. Phys. Rev. Lett..

[cit21] Jewell A. D., Tierney H. L., Sykes E. C. H. (2010). Gently lifting gold's herringbone reconstruction: Trimethylphosphine on Au(111). Phys. Rev. B: Condens. Matter Mater. Phys..

[cit22] Rossel F., Brodard P., Patthey F., Richardson N. V., Schneider W.-D. (2008). Modified herringbone reconstruction on Au(111) induced by self-assembled Azure A islands. Surf. Sci..

[cit23] Corso M., Schiller F., Fernández L., Cordón J., Ortega J. E. (2009). Electronic states in faceted Au(111) studied with curved crystal surfaces. J. Phys.: Condens. Matter.

[cit24] Crommie M. F., Lutz C. P., Eigler D. M. (1993). Confinement of Electrons to Quantum Corrals on a Metal Surface. Science.

[cit25] Crommie M. F., Lutz C. P., Eigler D. M., Heller E. J. (1995). WAVES ON A METAL SURFACE AND QUANTUM CORRALS. Surf. Rev. Lett..

[cit26] Stilp F. (2021). *et al.*, Very weak bonds to artificial atoms formed by quantum corrals. Science.

[cit27] Gomes K. K., Mar W., Ko W., Guinea F., Manoharan H. C. (2012). Designer Dirac fermions and topological phases in molecular graphene. Nature.

[cit28] Manoharan H. C., Lutz C. P., Eigler D. M. (2000). Quantum mirages formed by coherent projection of electronic structure. Nature.

[cit29] Freeney S. E., Slot M. R., Gardenier T. S., Swart I., Vanmaekelbergh D. (2022). Electronic Quantum Materials Simulated with Artificial Model Lattices. ACS Nanosci. Au.

[cit30] Slot M. R. (2017). *et al.*, Experimental realization and characterization of an electronic Lieb lattice. Nat. Phys..

[cit31] Kempkes S. N. (2019). *et al.*, Design and characterization of electrons in a fractal geometry. Nat. Phys..

[cit32] Kempkes S. N. (2019). *et al.*, Robust zero-energy modes in an electronic higher-order topological insulator. Nat. Mater..

[cit33] Kühnle A. (2009). Self-assembly of organic molecules at metal surfaces. Curr. Opin. Colloid Interface Sci..

[cit34] Dong L., Gao Z., Lin N. (2016). Self-assembly of metal–organic coordination structures on surfaces. Prog. Surf. Sci..

[cit35] Hu W. (2022). *et al.*, Engineering novel surface electronic states *via* complex supramolecular tessellations. Nanoscale.

[cit36] Piquero-Zulaica I. (2022). *et al.*, Engineering quantum states and electronic landscapes through surface molecular nanoarchitectures. Rev. Mod. Phys..

[cit37] Lobo-Checa J. (2009). *et al.*, Band Formation from Coupled Quantum Dots Formed by a Nanoporous Network on a Copper Surface. Science.

[cit38] Piquero-Zulaica I. (2017). *et al.*, Precise engineering of quantum dot array coupling through their barrier widths. Nat. Commun..

[cit39] Piquero-Zulaica I. (2019). *et al.*, Electron Transmission through Coordinating Atoms Embedded in Metal-Organic Nanoporous Networks. Phys. Rev. Lett..

[cit40] Ruben M. (2006). *et al.*, 2D Supramolecular Assemblies of Benzene-1,3,5-triyl-tribenzoic Acid: Temperature-Induced Phase Transformations and Hierarchical Organization with Macrocyclic Molecules. J. Am. Chem. Soc..

[cit41] Kampschulte L. (2008). *et al.*, Thermodynamical Equilibrium of Binary Supramolecular Networks at the Liquid–Solid Interface. J. Am. Chem. Soc..

[cit42] Li J., Gottardi S., Solianyk L., Moreno-López J. C., Stöhr M. (2016). 1,3,5-Benzenetribenzoic Acid on Cu(111) and Graphene/Cu(111): A Comparative STM Study. J. Phys. Chem. C.

[cit43] Klappenberger F. (2009). *et al.*, Dichotomous Array of Chiral Quantum Corrals by a Self-Assembled Nanoporous Kagomé Network. Nano Lett..

[cit44] Lyu L. et al. , Temperature-driven confinements of surface electrons and adatoms in a weakly interacting 2D organic porous network. 2023, Preprint at 10.48550/ARXIV.2307.06808

[cit45] Tusche C., Krasyuk A., Kirschner J. (2015). Spin resolved bandstructure imaging with a high resolution momentum microscope. Ultramicroscopy.

[cit46] Dendzik M., Bianchi M., Michiardi M., Sanders C. E., Hofmann P. (2016). Reconstruction-induced trefoil knot Fermi contour of Au(111). Phys. Rev. B.

[cit47] LaShell S., McDougall B. A., Jensen E. (1996). Spin Splitting of an Au(111) Surface State Band Observed with Angle Resolved Photoelectron Spectroscopy. Phys. Rev. Lett..

[cit48] Reinert F., Nicolay G. (2004). Influence of the herringbone reconstruction on the surface electronic structure of Au(111). Appl. Phys. A.

[cit49] El-Fattah A., Kher-Elden Z. M., Piquero-Zulaica M. A., de Abajo I., Ortega F. J. G., Graphene J. E. (2019). Free electron scattering within an inverted honeycomb lattice. Phys. Rev. B.

[cit50] Horcas I. (2007). *et al.*, WSXM : A software for scanning probe microscopy and a tool for nanotechnology. Rev. Sci. Instrum..

