# Supplementary material for: An organic array of quantum corrals modulated by the gold herringbone electronic superlattice
Source: Nanoscale. 2025 Mar 18;17(16):10314–23. doi: 10.1039/d5nr00148j (PMC12678045; doi:10.1039/d5nr00148j)
Supplement: NR-017-D5NR00148J-s001 [file NR-017-D5NR00148J-s001.pdf]

Supplementary Information (SI) for Nanoscale.
This journal is © The Royal Society of Chemistry 2025

# Supplementary Information for: Organic array of quantum corrals modulated by the gold herringbone electronic superlattice

*Jun Li (1)†, Ignacio Piquero-Zulaica (2,3,4)†, Stefano Gottardi (1), Mustafa A. Ashoush (5), Zakaria M. Abd El-Fattah (5,6)*, Leonid Solianyk (1), Jose Enrique Ortega (2,7,8), Johannes V. Barth (3), Juan Carlos Moreno-Lopez (9), Jorge Lobo-Checa (10,11)* and Meike Stöhr (1,12)**

(1) Zernike Institute for Advanced Materials, University of Groningen, Nijenborgh 3, 9747 AG Groningen, Netherlands

† J.L. and I.P.-Z. contributed equally to this work.

(2) Centro de Física de Materiales CSIC/UPV-EHU, Manuel Lardizabal 5, 20018 San Sebastian, Spain

(3) Physics Department E20, TUM School of Natural Sciences, Technical University of Munich, James-Franck-Straße 1, D-85748 Garching, Germany.

(4) IKERBASQUE, Basque Foundation for Science, Plaza Euskadi 5, 48009 Bilbao, Spain.

(5) Physics Department, Faculty of Science, Al-Azhar University, Nasr City, E-11884, Cairo, Egypt.

(6) Physics Department, Faculty of Science, Galala University, New Galala City, Suez, 43511, Egypt

(7) Departamento de Física Aplicada I, Universidad del País Vasco, 20018 San Sebastian, Spain

(8) Donostia International Physics Center (DIPC), Paseo Manuel de Lardizabal 4, E-20018 Donostia-San Sebastian, Spain.

(9) Institute of Solid State Physics, Vienna University of Technology, 1040 Vienna, Austria.

(10) Instituto de Nanociencia y Materiales de Aragón, CSIC-Universidad de Zaragoza, E-50009 Zaragoza, Spain

(11) Departamento Física de la Materia Condensada, Universidad de Zaragoza, E-50009 Zaragoza, Spain

(12) University of Applied Sciences of the Grisons, Pulvermühlestrasse 57, 7000 Chur, Switzerland

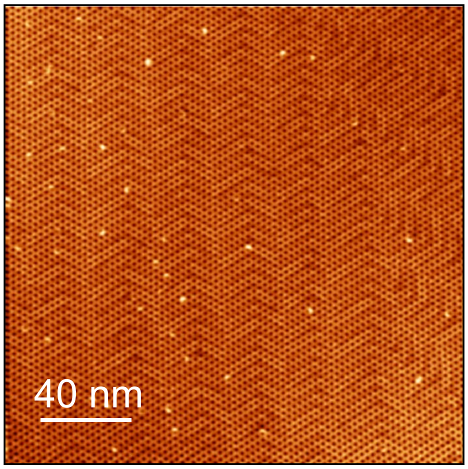


Figure S1: Large-scale overview STM image of the BTB quantum corral (QC) array on Au(111) ($V$ = -1.5 V; $I$ = 15 pA). The image has been smoothened in x and y directions in order to enhance the herringbone pattern below the QC array.

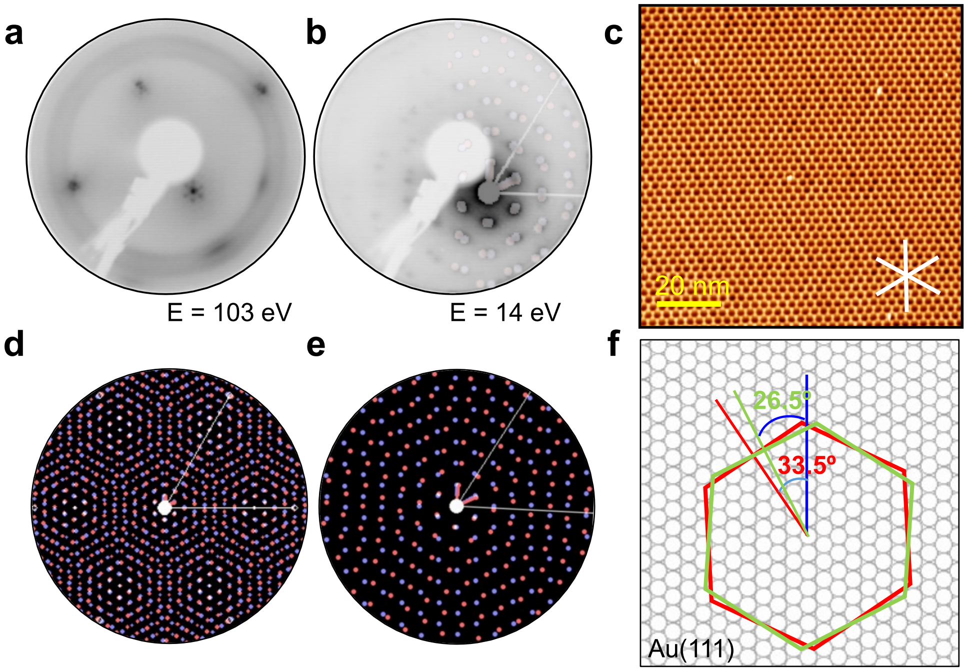


Figure S2: LEED patterns for the BTB network on Au(111) after post-deposition annealing at 400 K. (a) LEED image taken at 103 eV. The (0,0) and first order spots of the Au(111) surface are visible. Diffraction spots originating from the molecular superstructure are slightly visible around the (0,0) spot. (b) LEED image taken at 14 eV. Spots originating from the QC array are clearly visible. The simulated LEED pattern from (e) is superimposed on top. (c) Large-scale STM image of the same sample, highlighting the orientation of the QC array domain with respect to the main high symmetry directions of Au(111) ($V$ = -1.5 V; $I$ = 15 pA). (d, e) Simulated LEED pattern by using a matrix of (13, 7; -7, 6). (f) Schematic model showing the relative rotation between the two QC array domains and the Au(111) substrate.

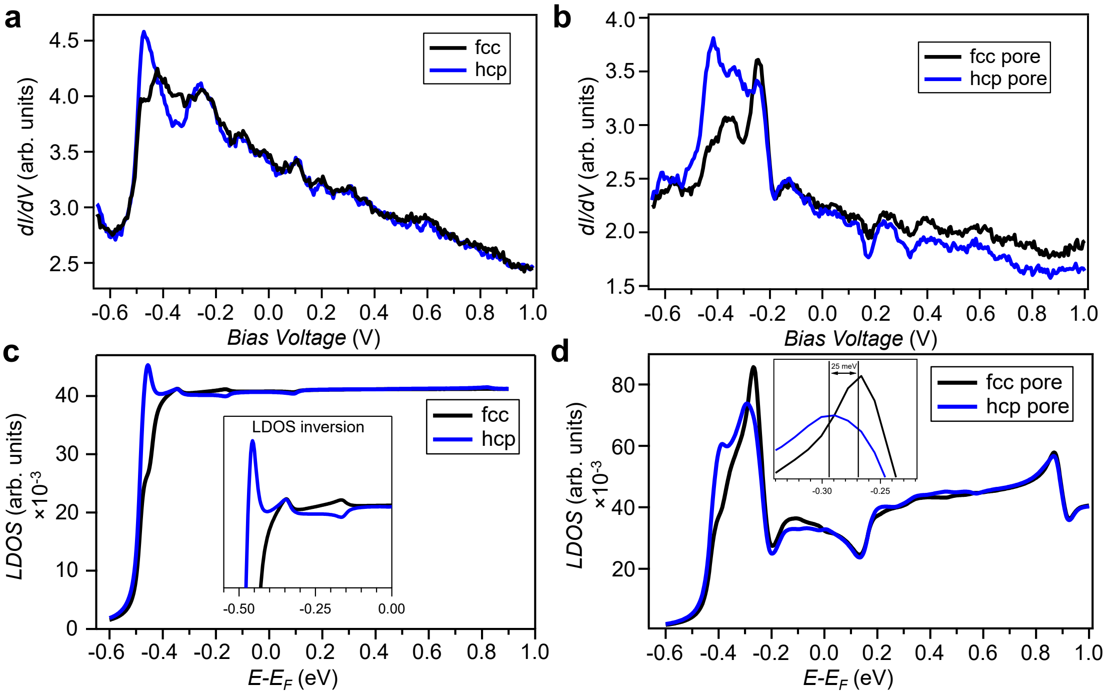


Figure S3: Scanning tunneling spectroscopy (STS) and EPWE LDOS simulations for the different sites of the herringbone reconstruction on bare Au(111) and the QC array/herringbone heterostructure. (a) STS spectra taken on the hcp (blue) and fcc (black) sites of the bare Au(111) substrate ($V_{set}$ = -700 mV; $I_{set}$ = 90 pA). (b) STS spectra taken in the center of a nanopore located on a hcp area (blue spectrum, position marked in Fig. 2d by a blue square) and on a nanopore located on a fcc area (black line, position marked in Fig. 2d by a black square) ($V_{set}$ = -700 mV; $I_{set}$ = 100 pA). (c) EPWE LDOS simulations for the herringbone reconstructed Au(111). The fcc spectrum is shown in black and the hcp one in blue. The inset highlights the LDOS intensity inversion between hcp and fcc curves at the herringbone gap. (d) EPWE LDOS simulations for the fcc nanopore center (black) and hcp nanopore center (blue). In the inset, the modulation of the *n=1* confined state is shown, where a ≈25 meV shift becomes evident at the top of the peak.

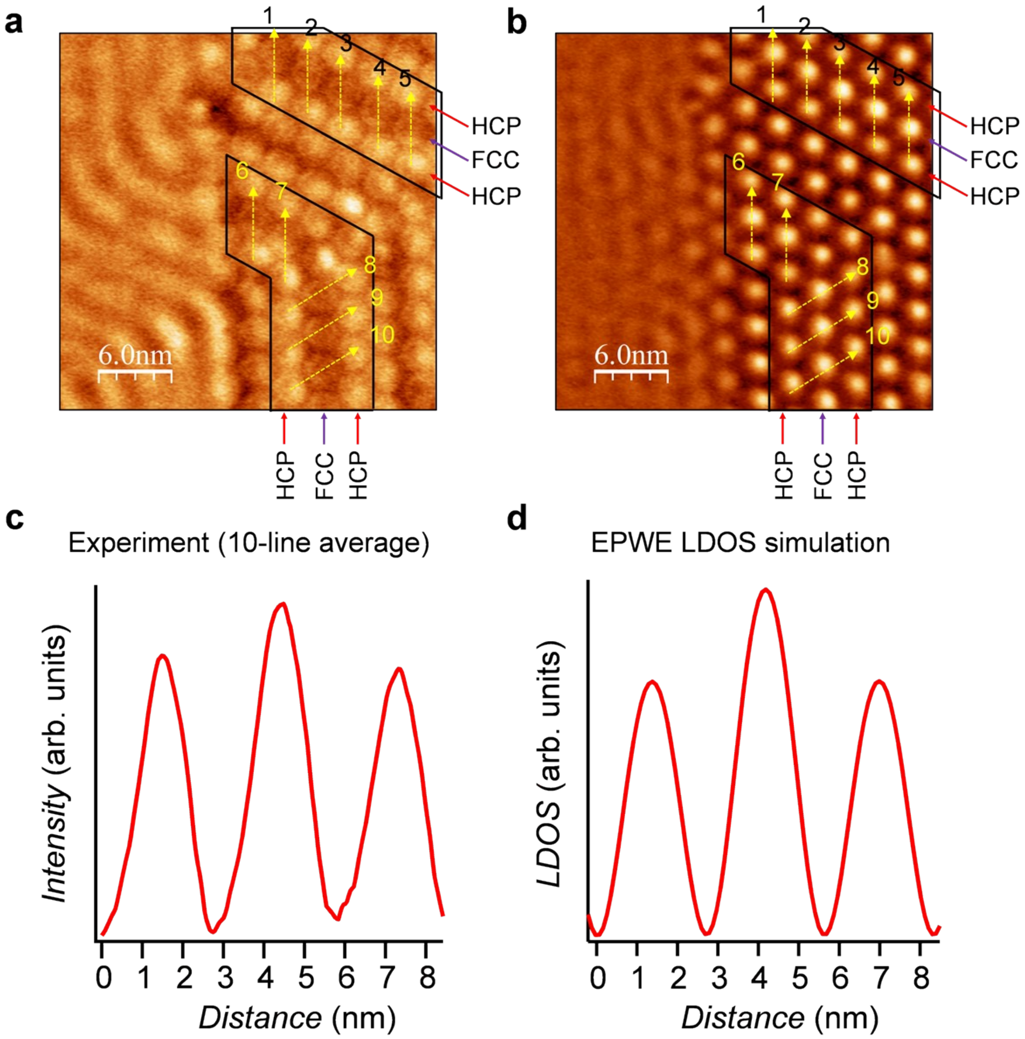


Figure S4: Conductance intensity line profiles traversing the hcp-fcc-hcp pores along straight segments of the herringbone reconstruction. (a) Constant current d$I$/d$V$ map taken at -0.40 V ($I$ = 90 pA) (same as Figure 2d of the main manuscript). This map allows us to identify hcp-fcc-hcp pores along straight segments of the herringbone reconstruction. (b) Constant current d$I$/d$V$ map taken at -0.25 V ($I$ = 90 pA) (same as Figure 2e of the main manuscript). The average of 10 line profiles traversing hcp-fcc-hcp pores is performed in this image at the indicated positions. This map shows the top of the $n = 1$ confined state intensity, which is modulated between fcc (more intense) and hcp regions. (c) Average experimental conductance line profile across hcp-fcc-hcp pores obtained over the 10 trajectories marked in (b). (d) EPWE simulated LDOS intensity line profile traversing the hcp-fcc-hcp pores taken along the dashed line in Figure 3g of the main manuscript. The agreement between (c) and (d) is excellent.

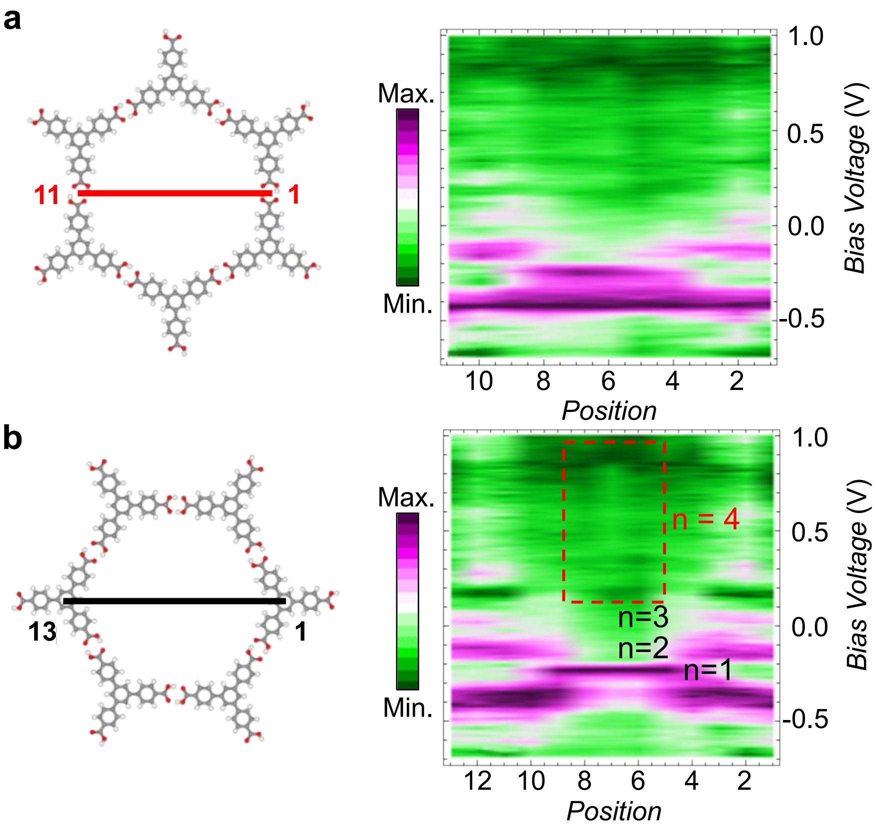


Figure S5: Normalized line STS spectra for the hcp type QC taken from side-to-side (a) and corner-to-corner (b). The n = 1, 2, 3 and 4 confined states are highlighted, the latter even with a red dashed rectangle and they can be compared with Fig. S6b showing the EPWE LDOS line simulations.

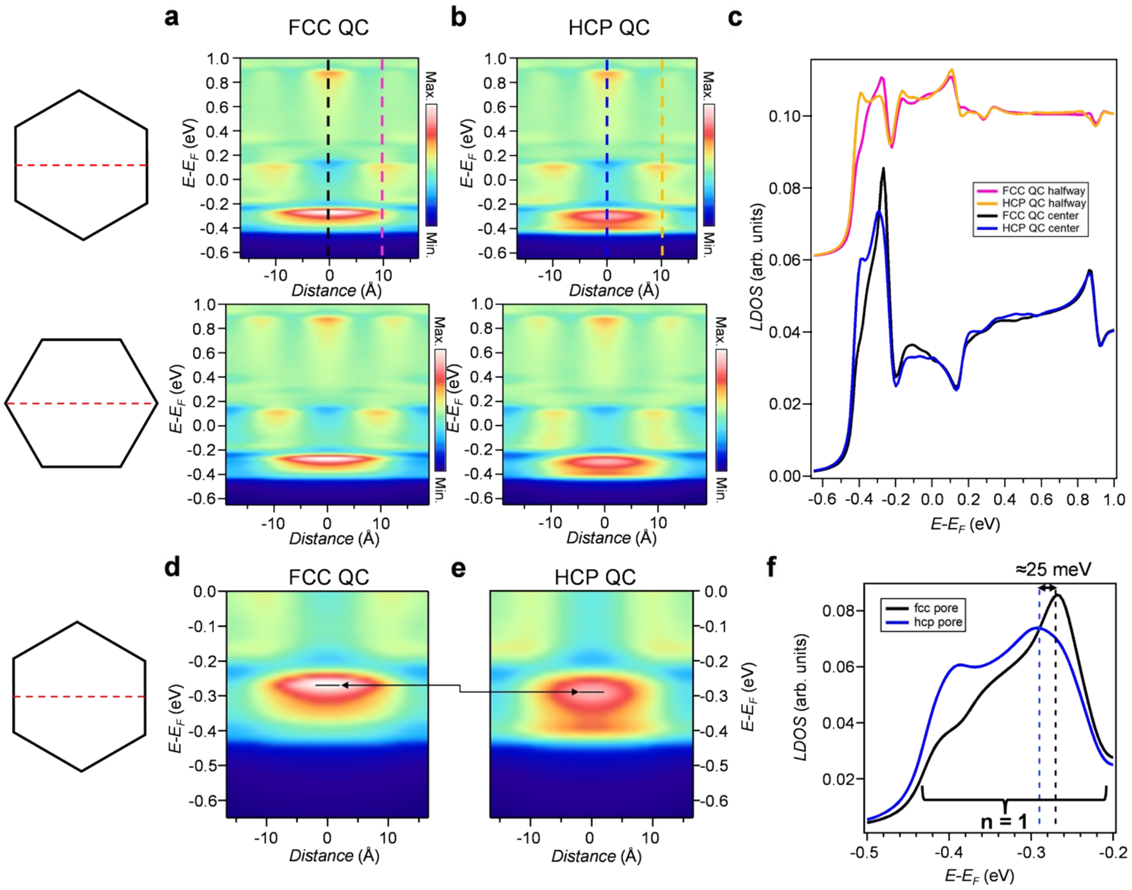


Figure S6: EPWE simulated LDOS line maps for fcc and hcp coupled QCs. (a) LDOS line simulations for the fcc QC taken side-to-side (top) and corner-to-corner (bottom). (b) LDOS line simulations for the hcp QC taken side-to-side (top) and corner-to-corner (bottom). (c) LDOS spectra comparing fcc and hcp QC center and halfway positions. These LDOS spectra were extracted from (a) and (b), as indicated by the dashed colored vertical lines. (d, e) Close up of the LDOS line simulations for the fcc and hcp QCs taken side-to-side on the $n = 1$ confined state. (f) LDOS spectra comparing fcc and hcp QC center positions focusing on the $n = 1$ confined state. The panels (d) to (f) show that the LDOS is more intense at the bottom of the first partially confined state in the hcp region, whereas the top of the $n = 1$ confined state peak becomes more intense in the fcc region and shifts ≈25 meV towards the Fermi level. This confirms the electronic modulation of the $n = 1$ partially localized state in coupled QCs by the gold herringbone electronic superlattice.

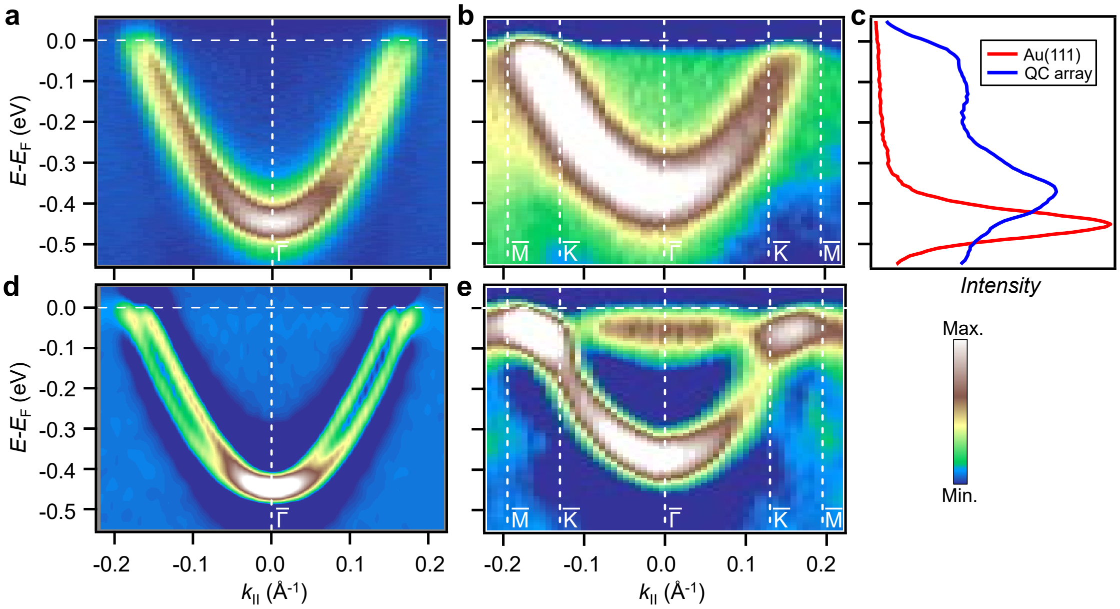


Figure S7: ARPES band structure comparing the pristine Au(111) surface with the QC array/herringbone heterostructure. (a, d) ARPES measurements of the pristine Au(111) Shockley surface state (raw data and second derivative, respectively). (b, e) ARPES measurements of the QC array modulated surface state in raw data and second derivative. (c) Energy distribution curves (EDCs) extracted at the $\bar{\Gamma}$ point comparing pristine Au(111) and QC array/herringbone heterostructure. A ≈75 meV shift of the band bottom upon network formation is clearly observed.
